# Multiple sclerosis management during the COVID-19 pandemic

**DOI:** 10.1177/1352458520948231

**Published:** 2020-08-10

**Authors:** Brandon P Moss, Kedar R Mahajan, Robert A Bermel, Kelsey Hellisz, Le H Hua, Timothy Hudec, Scott Husak, Marisa P McGinley, Daniel Ontaneda, Zhini Wang, Malory Weber, Paula Tagliani, Simón Cárdenas-Robledo, Ana Zabalza, Georgina Arrambide, Pere Carbonell-Mirabent, Marta Rodríguez-Barranco, Jaume Sastre-Garriga, Mar Tintore, Xavier Montalban, Morgan Douglas, Esther Ogbuokiri, Berna Aravidis, Jeffrey A Cohen, Ellen M Mowry, Kathryn C Fitzgerald

**Affiliations:** Mellen Center for Multiple Sclerosis, Cleveland Clinic, Cleveland, OH, USA; Mellen Center for Multiple Sclerosis, Cleveland Clinic, Cleveland, OH, USA; Mellen Center for Multiple Sclerosis, Cleveland Clinic, Cleveland, OH, USA; Mellen Center for Multiple Sclerosis, Cleveland Clinic, Cleveland, OH, USA; Lou Ruvo Center for Brain Health, Cleveland Clinic, Las Vegas, NV, USA; Mellen Center for Multiple Sclerosis, Cleveland Clinic, Cleveland, OH, USA; Mellen Center for Multiple Sclerosis, Cleveland Clinic, Cleveland, OH, USA; Mellen Center for Multiple Sclerosis, Cleveland Clinic, Cleveland, OH, USA; Mellen Center for Multiple Sclerosis, Cleveland Clinic, Cleveland, OH, USA; Department of Quantitative Health Sciences, Cleveland Clinic, Cleveland, OH, USA; Mellen Center for Multiple Sclerosis, Cleveland Clinic, Cleveland, OH, USA; Servei de Neurologia-Neuroimmunologia, Centre d’Esclerosi Múltiple de Catalunya, (CEMCAT), Vall d’Hebron Institut de Recerca, Vall d’Hebron Hospital Universitari, Universitat Autònoma de Barcelona, Barcelona, Spain; Servei de Neurologia-Neuroimmunologia, Centre d’Esclerosi Múltiple de Catalunya, (CEMCAT), Vall d’Hebron Institut de Recerca, Vall d’Hebron Hospital Universitari, Universitat Autònoma de Barcelona, Barcelona, Spain; Servei de Neurologia-Neuroimmunologia, Centre d’Esclerosi Múltiple de Catalunya, (CEMCAT), Vall d’Hebron Institut de Recerca, Vall d’Hebron Hospital Universitari, Universitat Autònoma de Barcelona, Barcelona, Spain; Servei de Neurologia-Neuroimmunologia, Centre d’Esclerosi Múltiple de Catalunya, (CEMCAT), Vall d’Hebron Institut de Recerca, Vall d’Hebron Hospital Universitari, Universitat Autònoma de Barcelona, Barcelona, Spain; Servei de Neurologia-Neuroimmunologia, Centre d’Esclerosi Múltiple de Catalunya, (CEMCAT), Vall d’Hebron Institut de Recerca, Vall d’Hebron Hospital Universitari, Universitat Autònoma de Barcelona, Barcelona, Spain; Servei de Neurologia-Neuroimmunologia, Centre d’Esclerosi Múltiple de Catalunya, (CEMCAT), Vall d’Hebron Institut de Recerca, Vall d’Hebron Hospital Universitari, Universitat Autònoma de Barcelona, Barcelona, Spain; Servei de Neurologia-Neuroimmunologia, Centre d’Esclerosi Múltiple de Catalunya, (CEMCAT), Vall d’Hebron Institut de Recerca, Vall d’Hebron Hospital Universitari, Universitat Autònoma de Barcelona, Barcelona, Spain; Servei de Neurologia-Neuroimmunologia, Centre d’Esclerosi Múltiple de Catalunya, (CEMCAT), Vall d’Hebron Institut de Recerca, Vall d’Hebron Hospital Universitari, Universitat Autònoma de Barcelona, Barcelona, Spain; Servei de Neurologia-Neuroimmunologia, Centre d’Esclerosi Múltiple de Catalunya, (CEMCAT), Vall d’Hebron Institut de Recerca, Vall d’Hebron Hospital Universitari, Universitat Autònoma de Barcelona, Barcelona, Spain; Division of Neuroimmunology and Neurological Infections, Johns Hopkins University, Baltimore, MA, USA; Division of Neuroimmunology and Neurological Infections, Johns Hopkins University, Baltimore, MA, USA; Division of Neuroimmunology and Neurological Infections, Johns Hopkins University, Baltimore, MA, USA; Mellen Center for Multiple Sclerosis, Cleveland Clinic, Cleveland, OH, USA; Division of Neuroimmunology and Neurological Infections, Johns Hopkins University, Baltimore, MA, USA; Division of Neuroimmunology and Neurological Infections, Johns Hopkins University, Baltimore, MA, USA

**Keywords:** COVID-19, SARS-CoV-2, multiple sclerosis, disease modifying therapies, health behaviors, healthcare delivery

## Abstract

**Background:**

People with multiple sclerosis (MS) may be at higher risk for complications from the 2019 coronavirus (COVID-19) pandemic due to use of immunomodulatory disease modifying therapies (DMTs) and greater need for medical services.

**Objectives:**

To evaluate risk factors for COVID-19 susceptibility and describe the pandemic’s impact on healthcare delivery.

**Methods:**

Surveys sent to MS patients at Cleveland Clinic, Johns Hopkins, and Vall d’Hebron-Centre d’Esclerosi Múltiple de Catalunya in April and May 2020 collected information about comorbidities, DMTs, exposures, COVID-19 testing/outcomes, health behaviors, and disruptions to MS care.

**Results:**

There were 3028/10,816 responders. Suspected or confirmed COVID-19 cases were more likely to have a known COVID-19 contact (odds ratio (OR): 4.38; 95% confidence interval (CI): 1.04, 18.54). In multivariable-adjusted models, people who were younger, had to work on site, had a lower education level, and resided in socioeconomically disadvantaged areas were less likely to follow social distancing guidelines. 4.4% reported changes to therapy plans, primarily delays in infusions, and 15.5% a disruption to rehabilitative services.

**Conclusion:**

Younger people with lower socioeconomic status required to work on site may be at higher exposure risk and are potential targets for educational intervention and work restrictions to limit exposure. Providers should be mindful of potential infusion delays and MS care disruption.

## Introduction

Coronavirus disease 2019 (COVID-19), caused by severe acute respiratory syndrome coronavirus 2 (SARS-CoV-2), is a pandemic with a high case fatality rate (534,062 deaths out of 11,425,209 confirmed cases as of 7 July 2020) and is associated with unprecedented changes to daily life and delivery of healthcare.^1^ People with multiple sclerosis (MS), an inflammatory demyelinating and neurodegenerative disorder treated with immunomodulatory disease modifying therapies (DMTs), may be at higher risk than the general public due to neurologic disability,^2^ the impact of DMTs on COVID-19 susceptibility and severity, and the effects of SARS-CoV-2 on MS disease activity. Moreover, many people with MS need access to regular medical services (infusions, physical therapy, occupational therapy, botulinum toxin injections for spasticity, and homecare services), which could be disrupted by changes to healthcare delivery as a result of the COVID-19 pandemic.

To better understand the impact of COVID-19 on the MS population, three large MS centers in the United States (US) and Europe, the Cleveland Clinic Mellen Center for MS Treatment and Research (CC), the Johns Hopkins MS Precision Medicine Center of Excellence (JH), and the Vall d’Hebron Centre d’Esclerosi Múltiple de Catalunya (CEMCAT), sent a comprehensive survey to their respective clinic populations. The goals of the survey and the current manuscript are to evaluate candidate risk factors for COVID-19 susceptibility and describe the impact of the pandemic on routine MS management in a large international MS clinical population. Here we report the results from the first 3028 participants.

## Materials and methods

### Study design

This is a multicenter observational study combining survey data with detailed demographics, MS disease characteristics, and a battery of patient-reported outcome measures from clinical registries maintained at each participating institution. Sites prospectively coordinated data collection to ensure that key survey and registry data were consistent across institutions.

This study was conducted in accordance with the International Conference on Harmonization Good Clinical Practice Guideline, the Declaration of Helsinki, and local ethical and legal requirements. Approval was obtained from the institutional review boards of each participating institution.

### Study population

Participants were recruited from the clinic populations of CC, JH, and CEMCAT. Surveys were sent to adults age 18 and older, followed by a provider from one of the participating MS centers, with a clinical diagnosis of MS confirmed by chart review. Surveys were sent by email or secure electronic medical record communication. Survey data were collected and managed using REDCap electronic data capture tools hosted at each institution.^3^ Responders reported whether they had confirmed COVID-19 (viral symptoms with a positive SARS-CoV-2 test result) or, if untested, whether a healthcare provider suspected they had COVID-19.

All three MS centers are members of Multiple Sclerosis Partners Advancing Technology and Health Solutions (MS PATHS), a learning health system sponsored by Biogen in collaboration with 10 healthcare institutions in the United States and Europe.^4^ MS PATHS sites use a tablet-based application to collect standardized patient data as part of routine clinical care.

### Study assessments

Survey questionnaires sent in April and May of 2020 collected core information regarding living situation, comorbidities, current DMT use, smoking and drug use, work and home exposure risks, travel, recent illness and symptoms, SARS-CoV-2 testing, and COVID-19 outcomes and treatments from all sites. Additional information about use of immunomodulatory therapies for conditions other than MS, health behaviors (social distancing, mask use, and number of weekly contacts outside the home), and the presence of a relapse or worsening MS symptoms associated with COVID-19 illness were collected by CC and JH. The impact of the pandemic on routine clinical care (delay, discontinuation, or modification of DMT use, and interruption of services) was collected by CC. A strict countrywide lockdown was in place for Spain at the time of survey completion, only allowing people to leave their homes to buy food and medicine, to work, and to care for minors, the elderly, and other vulnerable people. As a result, social distancing practices were not expected to vary widely, and questions about health behaviors were omitted from the CEMCAT survey.

Clinical practice registries contained demographics (age, sex, race, ethnicity, and indicators of socioeconomic status (SES)), information about MS disease history and treatment (disease duration, disease course, and current and prior DMTs), and measures of disability. Disability was assessed by the Expanded Disability Status Scale (EDSS), the most commonly used measure of MS-related disability,^5^ or the Patient Determined Disease Steps (PDDS), a validated self-report measure of MS disability strongly correlated with the EDSS.^6^ Indicators of SES included years of education and the Area Deprivation Index (ADI) for the US sites. The ADI is a composite measure that incorporates 17 different measures of SES derived using geo-coded addresses; nationwide indices range from 0 (least disadvantaged) to 100 (most disadvantaged).^7^

### Statistical methods and data analysis

Baseline demographics, disease characteristics, and key comorbidities were reported with descriptive statistics (mean and standard deviation for continuous variables and frequency and percentage for categorical variables). Differences between survey responders and non-responders; participants with and without suspicion of having COVID-19; and SARS-CoV-2 positive and negative cases were tested using logistic regression models. Predictors of non-adherence to health behaviors were assessed using univariate and multivariable-adjusted models performed with logistic regression methods. Similar analyses evaluated potential differences across sites via generalized linear models where appropriate. Statistical analysis was performed with the R software, version 3.6.2.^8^

## Results

Of 10,816 people sent a questionnaire, 3028 (28%) finished the survey. Survey responders were predominantly female (75%) and white (90%) with relapsing MS (72%). Across surveys, responders were older, more likely to be female, more likely to be non-Hispanic whites, and had higher SES compared to non-responders.

Table 1 summarizes characteristics of responders by presence or absence of SARS-CoV-2 testing. For all responders, both tested and untested, 2334 (77%) were on a DMT, 200 (7%) were taking an immunomodulatory therapy for a condition other than MS, 448 (15%) were current smokers or vapers, and 1376 (45%) had a medical comorbidity suspected to be a risk factor for more severe COVID-19 (obesity, hypertension, chronic obstructive pulmonary disease (COPD), diabetes, asthma, other chronic lung disease, coronary artery disease, stroke, chronic kidney disease, cancer, other inflammatory disease, or HIV). Of those with a medical comorbidity suspected to be a risk factor for more severe COVID-19, obesity (28.8%) and hypertension (19.1%) were most common. Contact with a known positive COVID-19 case was relatively uncommon (1.8%).

**Table 1.** Survey population by COVID-19 testing status.

	COVID-19 testing status		
	Not tested	Tested	*p* value
*n*	2938	90	
Age, mean (SD)	50.31 (12.09)	48.85 (12.42)	0.26
Male sex, *n* (%)	740 (25.2)	13 (14.4)	0.03
Race, *n* (%)			
White	2644 (90.0)	79 (87.8)	0.35
Black/African American	149 (5.1)	8 (8.9)	
Other	67 (2.3)	2 (2.2)	
Unknown	77 (2.6)	1 (1.1)	
Hispanic/Latino ethnicity	61 (2.1)	1 (1.1)	0.80
Area Deprivation Index, mean (SD)^a^	41.03 (25.14)	41.48 (25.21)	0.88
High school education or less, *n* (%)^a^	316 (14.4)	7 (9.2)	0.27
Disease duration, mean (SD)	16.40 (11.14)	17.50 (12.00)	0.21
RRMS, *n* (%)	2118 (72.1)	70 (77.8)	0.37
PDDS, mean (SD)^a^	2.07 (2.22)	1.68 (2.01)	0.56
EDSS, mean (SD)^b^	2.78 (2.00)	2.19 (2.43)	0.88
DMT, *n* (%)			0.76
Infusion	943 (33.3)	31 (34.4)	
Injectable	444 (15.7)	10 (11.1)	
None	669 (23.6)	25 (27.8)	
Oral	715 (25.3)	22 (24.4)	
Other	59 (2.1)	2 (2.2)	
Not reported	97 (3.3)	0 (0.0)	
Other immunotherapy, *n* (%)^a^	183 (6.2)	17 (18.9)	<0.001
Any comorbidity, *n* (%)^c^	1322 (45.0)	54 (60.0)	0.007
Current smoker or vaper, *n* (%)^a^	433 (14.7)	15 (16.7)	0.72
Working on site, *n* (%)^a^	348 (15.9)	18 (23.7)	0.10
Positive COVID-19 test, *n* (%)	–	17	
Contact with positive case, *n* (%)	39 (1.7)	15 (16.7)	<0.001
Positive household contact, *n* (%)	99 (3.4)	12 (13.3)	<0.001

SD: standard deviation; RRMS: relapsing-remitting multiple sclerosis; PDDS: Patient Determined Disease Steps; EDSS: Expanded Disability Status Scale; DMT: disease modifying therapies.

a Data available in CC and JH surveys (*n* = 2270).

b Data available in CEMCAT survey (*n* = 758).

c Any comorbidity denotes having obesity, hypertension, COPD, diabetes, asthma, other lung diseases, coronary artery disease, stroke, chronic kidney disease, cancer, other inflammatory diseases, or HIV.

Of 90 (3.0%) who reported having been tested for SARS-CoV-2, 17 (18.8%) reported having a positive test. Among those tested for SARS-CoV-2, there was a lower proportion of men (14.4% vs. 25.2%), higher proportion of people on immunomodulatory therapies for conditions other than MS (18.9% vs. 6.2%), and higher proportion of people with medical comorbidities (60.0% vs. 45.0%) compared to untested individuals.

There were 77 (2.5%) cases of suspected or confirmed COVID-19. Factors associated with suspected or confirmed COVID-19 are summarized in Table 2. Suspected or confirmed cases were more likely to have contact with someone with known COVID-19 (odds ratio (OR): 4.38; 95% confidence interval (CI): 1.04, 18.54) or to live with someone with COVID-19 (OR: 13.94; 95% CI: 7.84, 24.80). For the US residents, there was also a higher proportion of African Americans (15.6% vs. 6.8%) and people of lower SES (ADI: 45.8 vs. 40.9), but these did not rise to the level of statistical significance (OR 2.56; 95% CI: 0.97, 6.77 and OR 1.05; 95% CI: 0.90, 1.22, respectively). Suspected or confirmed COVID-19 was more prevalent for Spanish than the US residents (OR: 3.78; 95% CI: 2.31, 6.17).

**Table 2.** Factors associated with suspected or confirmed COVID-19^a^ in people with MS.

	No COVID-19	Suspected or confirmed COVID-19^a^	OR (95% CI) for suspected or confirmed COVID-19^b^
*N*	2951	77	
Site, *n* (%)			
Cleveland Clinic	1557 (52.8)	26 (33.8)	1.00 (ref)
Johns Hopkins	681 (23.1)	6 (7.8)	0.53 (0.22, 1.29)
CEMCAT	713 (24.2)	45 (58.4)	**3.78 (2.31, 6.17)**
Age, mean (SD)^d^	50.36 (12.12)	46.79 (10.84)	0.85 (0.63, 1.15)
Male sex, *n* (%)	738 (25.5)	15 (19.7)	0.62 (0.35, 1.10)
Race, *n* (%)			
White	2652 (89.9)	71 (92.2)	1.00 (ref)
Black/African American	152 (5.2)	5 (6.5)	2.56 (0.97, 6.77)
Other	68 (2.3)	1 (1.3)	1.16 (0.15, 8.75)
Unknown	78 (2.6)	0 (0.0)	–
Hispanic/Latino ethnicity, *n* (%)^c^	62 (2.1)	0 (0.0)	–
Working on site, *n* (%)^c^	360 (16.1)	6 (18.8)	1.14 (0.46, 2.79)
Area Deprivation Index, mean (SD)^d,c^	40.97 (25.13)	45.91 (25.75)	1.05 (0.90, 1.22)
High school education or less, *n* (%)^c^	323 (14.4)	0 (0.0)	
RRMS, *n* (%)	2131 (72.2)	57 (74.0)	1.04 (0.62, 1.75)
PDDS/EDSS, mean (SD)	2.24 (2.19)	2.30 (1.97)	0.95 (0.85, 1.07)
Disease duration, mean (SD)	16.43 (11.19)	16.60 (10.15)	1.15 (0.93, 1.41)
DMT, *n* (%)			
Injectable	438 (15.4)	16 (21.1)	1.00 (ref)
Infusion	949 (33.4)	25 (32.9)	0.93 (0.49, 1.78)
None	684 (24.1)	10 (13.2)	0.52 (0.23, 1.17)
Oral	714 (25.1)	23 (30.3)	1.01 (0.52, 1.95)
Other	59 (2.1)	2 (2.6)	0.79 (0.18, 3.58)
Number of times leaving home per week, mean (SD)^c^	1.52 (3.10)	2.47 (11.44)	**1.07 (1.01, 1.14)**
Number of times household contacts leaving home per week, mean (SD)^c^	1.42 (2.51)	1.25 (2.16)	0.97 (0.81, 1.15)
Not adhering to social distancing recommendations, *n* (%)^c^	432 (19.3)	8 (25.0)	1.31 (0.58, 2.94)
Viral symptoms, *n* (%)	782 (26.5)	71 (92.2)	66.09 (27.84, 156.88)
Contact with positive case, *n* (%)	38 (1.7)	16 (20.8)	**4.38 (1.04, 18.54)**
Positive household contact, *n* (%)	83 (2.8)	28 (36.4)	**13.94 (7.84, 24.80)**
Any comorbidity, *n* (%)^e^	1339 (49.7)	37 (50.0)	**1.83 (1.09, 3.07)**
Current smoker or vaper, *n* (%)	432 (15.7)	16 (21.1)	1.45 (0.82, 2.57)

SD: standard deviation; CI: confidence interval; RRMS: relapsing-remitting multiple sclerosis; PDDS: Patient Determined Disease Steps; EDSS: Expanded Disability Status Scale; DMT: disease modifying therapies.

a Denotes individuals who were confirmed positive cases and those who were suspected by a healthcare professional of ever having COVID-19 but were never tested.

b OR are adjusted for location of suspected or confirmed COVID-19 case. Because of the relatively limited number of cases and cases were not distributed with equal frequency across centers, we did not perform extensive multivariable-adjusted analyses.

c Data for these variables were pooled from JH and CC surveys only (*n* = 2270, 32 with suspected or confirmed COVID-19).

d OR for age represents a per 15-year increase; OR for ADI represents a per 10 percentile increase in deprivation.

e Any comorbidity denotes having obesity, hypertension, COPD, diabetes, asthma, other lung diseases, coronary artery disease, stroke, chronic kidney disease, cancer, other inflammatory diseases, or HIV.

For the 2270 (75%) responders who were US residents, the majority were strictly self-isolating, 1830 (81%), only leaving home to get basic necessities or to go to work if they could not work from home; left home infrequently (mean 1.52 (standard deviation (SD): 3.37)); and wore a mask when out in public, 1329 (59%). We also conducted analyses assessing predictors of non-adherence to health behaviors (social distancing, mask use in public, and limiting number of weekly contacts outside the home) (Table 3). In multivariable-adjusted models, individuals having to work on site, with lower education level, and residing in more socioeconomically disadvantaged areas were less likely to be following social distancing guidelines. Older individuals were more likely to follow social distancing guidelines. Predictors of social distancing were similar when stratified by site (CC vs. JH).

**Table 3.** Factors associated with differences in social distancing behavior.

	Strictly self-isolating	At least some in-person socializing	OR (95% CI) for at least some in-person socializing	
			Univariate model	Multivariable-adjusted model^a^
*N*	1830	440		
Age, mean (SD)^b^	52.44 (12.06)	50.01 (11.85)	**0.78 (0.68, 0.89)**	0.80 (0.65, 1.00)
Male sex	405 (22.6)	115 (26.9)	1.26 (0.99, 1.60)	1.33 (0.99, 1.79)
Race				
White	1584 (86.6)	385 (87.5)	1.00 (ref)	1.00 (ref)
Black/African				
American	127 (6.9)	30 (6.8)	0.97 (0.64, 1.47)	1.06 (0.65, 1.74)
Other	53 (2.9)	13 (3.0)	1.01 (0.54, 1.87)	0.99 (0.43, 2.28)
Unknown	66 (3.6)	12 (2.7)	0.75 (0.40, 1.40)	1.35 (0.60, 3.01)
Hispanic/Latino ethnicity	53 (3.0)	9 (2.0)	0.70 (0.34, 1.43)	0.62 (0.25, 1.57)
Working on site	239 (13.1)	127 (28.7)	**2.70 (2.11, 3.46)**	**1.92 (1.41, 2.64)**
Area Deprivation Index, mean (SD)^b^	39.56 (24.92)	47.17 (25.13)	**1.13 (1.08, 1.17)**	**1.13 (1.07, 1.20)**
High school education or less	238 (13.0)	85 (19.3)	**1.60 (1.22, 2.10)**	**1.54 (1.10, 2.17**)
RRMS	1272 (71.0)	309 (72.5)	1.07 (0.85, 1.36)	1.08 (0.78, 1.50)
PDDS	2.09 (2.23)	1.91 (2.14)	0.96 (0.92, 1.01)	1.02 (0.94, 1.10)
Disease duration	17.86 (11.52)	16.32 (10.97)	**0.88 (0.80, 0.97)**	1.02 (0.89, 1.18)
DMT				
Injectable	242 (14.0)	40 (9.1)	1.00 (ref)	1.00 (ref)
Infusion	609 (35.1)	162 (36.9)	**1.61 (1.10, 2.35)**	1.30 (0.82, 2.06)
None	451 (26.0)	94 (21.4)	1.26 (0.84, 1.88)	1.47 (0.91, 2.36)
Oral	408 (23.5)	134 (30.5)	**1.99 (1.35, 2.93)**	**1.75 (1.10, 2.77)**
Other	24 (1.4)	9 (2.1)	2.27 (0.98, 5.23)	**2.86 (1.02, 8.01)**
Wearing a mask when leaving the house	1086 (59.3)	243 (55.2)	0.85 (0.69, 1.04)	**0.64 (0.49, 0.85)**
Number of times leaving the house per week	1.26 (2.76)	2.58 (4.93)	**1.11 (1.07, 1.14)**	**1.12 (1.08, 1.17)**
Any comorbidity^c^	967 (59.8)	242 (60.2)	1.02 (0.81, 1.27)	1.03 (0.78, 1.35)
Current smoker or vaper	255 (15.4)	87 (20.7)	**1.44 (1.09, 1.88)**	1.09 (0.78, 1.53)

SD: standard deviation; CI: confidence interval; RRMS: relapsing-remitting multiple sclerosis; PDDS: Patient Determined Disease Steps; EDSS: Expanded Disability Status Scale; DMT: Disease Modifying Therapies.

a Multivariable OR is adjusted for all factors included in the table.

b OR for age represents a per 15-year increase; OR for ADI represents a per 10 percentile increase in deprivation.

c Any comorbidity denotes having obesity, hypertension, COPD, diabetes, asthma, other lung diseases, coronary artery disease, stroke, chronic kidney disease, cancer, other inflammatory diseases, or HIV.

Of the 90 suspected or confirmed COVID-19 cases, 5 (5.6%) were hospitalized (1 from CC; 4 from CEMCAT). 67 (74.4%) were recovering or fully recovered at the time of the survey, while 3 (3.3%) had worsening symptoms or were recovering with complications.

Table 4 summarizes changes in MS management that occurred as a result of the COVID-19 pandemic and incorporates results pooled from JH and CC (*n* = 2270). 100 (4.4%) survey responders reported changes to their therapy plans. By far, the most common type of medication changes were delays in infusions (71.9%). 94.6% cite preventing COVID-19 as the reason for delay. Only half (51.2%) were advised to delay their infusion therapy by a healthcare provider.

**Table 4.** Changes in MS management occurring as a result of COVID-19.

Characteristic	*N* (%)
Disruption to rehabilitative therapy	355 (15.5)
Disruption to homecare services	51 (2.2)
Changed or adjusted MS therapy	100 (4.4)
Type of medication change	
Changed	8 (8.2)
Delay	71 (73.2)
Stopped	18 (18.6)

MS: multiple sclerosis.

With respect to effects on MS care, 351 (15.5%) reported a disruption to rehabilitative therapy (physical therapy, occupational therapy, or botulinum toxin injections), while 51 (2.2%) reported a disruption to homecare services. Those reporting disruption to rehabilitative therapy or homecare services were more likely to be older (mean age 55.7 vs. 51.3 years; *p* < 0.001), have progressive MS (47.9% vs. 25.2%; *p* < 0.001), have a walking aid (46.4% vs. 18.9% cane use; *p* < 0.001), and have a comorbid condition (65.2% vs. 58.8%; *p* = 0.04).

## Discussion

The COVID-19 pandemic presents a unique challenge to people with MS given the immune pathogenesis of MS, use of treatments with immunomodulatory actions, and need for access to regular medical services.^9^ In evaluating factors associated with suspected or confirmed COVID-19 illness, only contact with someone with known COVID-19 at home or in the community was significant, highlighting the importance of health behaviors to prevent infection.

Overall, there was good compliance with reported social distancing measures, with 83% of participants reporting strict self-isolation. However, individuals required to work on site, with lower education level, and residing in more socioeconomically disadvantaged areas were less likely to report following social distancing guidelines, whereas older individuals were more likely to report following social distancing guidelines. Younger people with a lower education level may be an important target for educational outreach regarding health risks of COVID-19 and appropriate health behaviors. Because these individuals had fewer opportunities to work from home, clinician support for work restrictions may be important for people with MS who lack job flexibility, especially if they have risk factors for severe COVID-19 illness as defined in smaller studies of MS populations^2^ and larger studies of non-MS populations.^10–12^

With regard to practice management, most individuals continued DMT dosing without intentional delays or discontinuations, consistent with National MS Society and MS International Federation guidelines.^13,14^ However, 5% of survey responders reported changes to their therapy plans, most commonly delays in infusions. While most of these individuals were advised to delay their infusions, a large portion delayed their infusions for other reasons, possibly due to their own concerns about treatment during the COVID-19 pandemic and possibly due to interruptions of care. There was also a notable disruption of rehabilitative therapy and homecare services among survey responders. These findings are consistent with reports of disruptions in health services for other noncommunicable diseases.^15^ Interruptions to these services could have downstream consequences for MS-related outcomes and other aspects of health-related quality of life.

The relatively low proportion of tested individuals, potential for false negative polymerase chain reaction (PCR), and small number of cases makes estimation of COVID-19 prevalence and prevalence of adverse disease-related outcomes difficult, and, most likely, our survey missed those with the most severe outcomes. Nevertheless, of the 90 suspected or confirmed COVID-19 cases, there were few hospitalizations, and most reported having recovered or being fully recovered at the time of the survey. Larger ongoing registry studies will evaluate predictors of COVID-19 outcomes in people with MS.

Our study has certain limitations. As with any observational study, our survey is subject to ascertainment bias. Differences in the demographic characteristics of responders versus non-responders demonstrate some degree of ascertainment bias. In addition, more severe illness or hospitalization could have prevented prospective participants from responding. Even so, our findings were similar to a registry study of 11,672 individuals in the CC population at large, where men, African Americans, older individuals, and those with known COVID-19 exposure were at higher risk of testing positive for COVID-19.^16^

Ongoing surveys will facilitate case ascertainment and serve as a vehicle for future studies of change in behaviors over time and effects of the pandemic on patient-reported outcomes and MS disease management. Longitudinal follow-up will also allow assessment of the degree to which disruptions of care affect disease activity or severity independent of COVID-19.

## Conclusion

Our findings suggest that exposure risks are the driver of COVID-19 infection in the MS population. Younger people with lower SES required to work on site may be at higher risk for exposure and are potential targets for educational intervention and clinician support for work restrictions to limit exposure. Providers should be mindful of potential delays of infusion therapies and disruption of MS care more generally.
